# Loss of Neuroligin3 specifically downregulates retinal GABA_Aα_2 receptors without abolishing direction selectivity

**DOI:** 10.1371/journal.pone.0181011

**Published:** 2017-07-14

**Authors:** Mrinalini Hoon, Vidhyasankar Krishnamoorthy, Tim Gollisch, Bjoern Falkenburger, Frederique Varoqueaux

**Affiliations:** 1 Department of Molecular Neurobiology, Max Planck Institute of Experimental Medicine, Göttingen, Germany; 2 Deutsche Forschungsgemeinschaft Research Center for Molecular Physiology of the Brain, Göttingen, Germany; 3 Department of Ophthalmology, University Medical Center Göttingen, Göttingen, Germany; 4 Bernstein Center for Computational Neuroscience Göttingen, Göttingen, Germany; 5 Department of Neurology RWTH Aachen University, Aachen, Germany; 6 Department of Fundamental Neurosciences, University of Lausanne, Lausanne, Switzerland; University of Sydney, AUSTRALIA

## Abstract

The postsynaptic adhesion proteins Neuroligins (NLs) are essential for proper synapse function, and their alterations are associated with a variety of neurodevelopmental disorders. It is increasingly clear that each NL isoform occupies specific subsets of synapses and is able to regulate the function of discrete networks. Studies of NL2 and NL4 in the retina in particular have contributed towards uncovering their role in inhibitory synapse function. In this study we show that NL3 is also predominantly expressed at inhibitory postsynapses in the retinal inner plexiform layer (IPL), where it colocalizes with both GABA_A_- and glycinergic receptor clusters in a 3:2 ratio. In the NL3 deletion-mutant (knockout or KO) mouse, we uncovered a dramatic reduction of the number of GABA_A_α2-subunit containing GABA_A_ receptor clusters at the IPL. Retinal activity was thereafter assessed in KO and wild-type (WT) littermates by multi-electrode-array recordings of the output cells of retina, the retinal ganglion cells (RGCs). RGCs in the NL3 KO showed reduced spontaneous activity and an altered response to white noise stimulation. Moreover, upon application of light flashes, the proportion of cells firing at light offset (OFF RGCs) was significantly lower in the NL3 KO compared to WT littermates, whereas the relative number of cells firing at light onset (ON RGCs) increased. Interestingly, although GABA_A_α2-bearing receptors have been related to direction-selective circuits of the retina, features of direction selective-retinal ganglion cells recorded remained unperturbed in the NL3 KO. Together our data underscore the importance of NL3 for the integrity of specific GABA_A_ergic retinal circuits and identifies NL3 as an important regulator of retinal activity.

## Introduction

Neuroligins (NL1-4) are postsynaptic transmembrane adhesion molecules crucial for synapse maturation and function [1, 2]. NL3 and NL4 have been given particular importance as their mutations in humans have been causally linked to cases of autism [3–5]. Accordingly, the roles of NL3 and NL4 have been investigated in diverse neural networks using NL3 and NL4 deletion-mutant mice [1, 6–8]. NL4 regulates glycinergic synapse receptor clustering and function in retinal circuits [9] and GABAergic synapse function in hippocampal circuits [10]. In contrast, little is known about NL3 distribution and function in neural circuits. NL3 is widely expressed in the CNS [1] and has been reported at both excitatory and inhibitory postsynapses of cultured hippocampal neurons [11] and in the cerebellum [8]. Initial studies failed to uncover significant changes in excitatory or inhibitory transmission in the cortex and the hippocampus of the NL3 deletion-mutant mouse [6], but more recently subtle alterations in mGluR1-mediated signaling and excitatory transmission have been reported in the cerebellum of the NL3 deletion-mutant mouse [8]. Moreover alterations in tonic endocannabinoid signaling and inhibitory transmission have been uncovered in the hippocampus of the NL3 deletion-mutant mouse [12].

These observations encouraged us to investigate NL3 distribution and both the morphological and functional alterations subsequent to NL3 deletion in the retina, another neural model circuit with stereotyped connectivity and well-defined, stratified synaptic connections with a direct functional correlate [13, 14]. Previous studies in the retina of NL deletion-mutant mice have unraveled the importance of NL2 and NL4 for the functional integrity of inhibitory signaling and synapse function [9, 15]. In the present study, we show that NL3 is expressed at retinal GABAergic and glycinergic postsynapses, and that it is essential for the integrity of a subset of GABAergic postsynapses that regulate distinct features of retinal and visual function.

## Materials and methods

The gene targeting strategy used to generate NL3 deletion-mutant (KO) mouse on a C57BL/6 background has been described previously [1]. All experiments were performed on 8 to 12 week-old age-matched littermate wild-type (WT) and NL3 KO mice. Four to five mice were housed per cage in a room with a 12-h light–dark cycle with ad libitum access to food and water. Cages were changed once a week. The animal health status was controlled daily by animal caretakers as well as by a veterinarian. Systematic health monitoring was carried out quarterly according to FELASA recommendations. All experiments were performed in compliance with the guidelines for the welfare of experimental animals issued by the Federal Government of Germany, the NIH, and the Max Planck Society. Animal protocols were approved by the Institutional Animal Care and Use Committee of the Max Planck Institute of Experimental Medicine.

### Immunohistochemistry

Animals were deeply anesthetized with Isofluran (DeltaSelect) and decapitated. Eyes were quickly removed, lens dissected out and eyecups immersed in 2% paraformaldehyde in 0.1 M phosphate buffer, pH 7.4 (PB) for 5 min (for GABA and glycine receptor labeling) or 20 min followed by rinses in PB. Retinae were then isolated and cryoprotected overnight in 30% sucrose in PB. Alternating pieces of WT and KO retinae were frozen on top of each other in tissue freezing medium (Leica) and sectioned vertically at 14 μm at the cryostat. For immunolabeling, sections were preincubated for 1 hr in PB containing 0.2% gelatin and 0.1% Triton X-100 (PGT), incubated overnight in primary antibodies in PGT followed by a 1 hr incubation in secondary antibodies conjugated to Alexa dyes (1:2,000, Molecular Probes) in PGT. The antibodies used in this study were: rabbit polyclonal anti-NL3 (1:3,000;[1]), guinea-pig polyclonal anti-GABA_A_α1, anti-GABA_A_α2, anti-GABA_A_α3 and anti-GABA_A_γ2 subunits (1:10,000; 1:5000; 1:4,000; and 1:2,500, respectively;[16]; kindly provided by J.M. Fritschy, Zürich, Switzerland), mouse monoclonal anti-glycine receptor (GlyR) subunits (pan; mAb4a, 1:500; Synaptic Systems) or specifically anti-GlyRα1 (mAb2b, 1:500; Synaptic Systems), polyclonal goat anti-GlyRα2 and anti-GlyRα3 (both, 1:400, Santa Cruz), rabbit polyclonal anti-GlyRα4 (1:500, Chemicon), mouse monoclonal anti-PSD95 (1:1,000, Abcam), mouse monoclonal anti-gephyrin (clone 3B11, 1:1,000, Synaptic Systems); mouse monoclonal anti-PKCα (1:1,000, Biodesign), rabbit polyclonal anti-neurokinin 3 receptor (NK3r) (a gift from E. Grady)[17], mouse anti-tyrosine hydroxylase (TH) (1:2,000, Chemicon), mouse anti-bassoon (1:1,000, Stressgen), rabbit anti-vesicular acetylcholine transporter (VAChT) (1:2,000, Synaptic Systems), and mouse anti-GAD67 (1:1000, Chemicon).

### Imaging and quantification

Confocal images were acquired on an inverted TCS-SP2 confocal laser-scanning microscope (Leica Microsystems), with a 63X oil-immersion objective (N.A. 1.4) and a digital zoom factor of 4 for quantifications. To allow for comparisons, gain and offset were kept constant upon imaging of a given labeling. The AnalySIS software (Olympus) was used for image processing and puncta density analysis as follows: an open filter was applied to smoothen images; next, a separation filter was used to discern small fluorescent clusters (typically individual puncta were ≥ 0.5 μm), likely corresponding to synaptic puncta. All objects whose intensity was above background (gray value of 50 set as background) were counted within the entire thickness of the retinal inner plexiform layer (IPL). For estimation of pixel density, pixels above background were divided by the total number of pixels in the same field. For colocalization studies, single fluorescent puncta were contoured manually in a given channel, the resulting mask superimposed on the complementary channel, and the number of colocalized puncta determined manually [18]. To account for the number of random associations between two markers, the mask was superimposed on the complementary channel image, which had been flipped horizontally. The number of colocalized puncta with the flipped channel, thus, related to the number of random colocalizations between the two markers and was subtracted from the initial numbers to represent the true rate of overlap.

### Multi-electrode recordings

We investigated the effects of NL3 deletion on retinal ganglion cell activity by recording spiking responses from isolated retinas of KO and WT mice with multi-electrode arrays, similar to previously described recordings [19, 20]. These experiments were performed “blindly”, that is, the group of each individual animal (KO or WT) was reported to the experimenters only after experiments and analyses were completed. Prior to experiments, animals were dark-adapted for at least 30 min. Preparation of retinas was performed under infrared illumination, using a stereomicroscope equipped with night-vision goggles. Isolated retina pieces were placed ganglion cell-side-down onto multi-electrode arrays (Multichannel Systems, 60 electrodes, 10 μm electrode size) and held in place by a dialysis membrane stretched tightly across a plastic holder. During recordings, the retina was perfused at about 5 ml/min with oxygenated (95% O_2_ and 5% CO_2_) Ames’ medium, containing 22 mM NaHCO_3_ to keep a pH level of 7.4 and supplemented with a total of 10 mM glucose. The Ames’ medium was heated through an inline heater to maintain a constant temperature of the retina of around 33°C—35°C. Spikes were extracted from the recorded voltage traces by a custom-made spike sorting program, based on maximum-likelihood fitting of a Gaussian mixture model [21]. Only units with well-sorted spikes and a clear refractory period were used for further analysis. Visual stimuli were presented by projecting a computer-controlled, gamma-corrected CRT monitor (100 Hz refresh rate, 800x600 pixels, 6 μm pixel size on the retina) onto the retina with standard optics. The mean light level of all stimuli was in the photopic range with an intensity of either 5.1 or 9.1 mW/m^2^.

Recorded ganglion cells were classified as ON-, OFF-, or ON-OFF-type according to the evoked spiking responses under alternating steps in full-field light intensity at 100% contrast. Spontaneous activity was assessed under constant illumination at mean light intensity for 2 min by counting the total number of spikes and dividing by the observation time. Receptive fields were measured by stimulating with binary spatio-temporal white noise, arranged on a rectangular grid (square size 60 μm, updated 50 Hz, 100% contrast), computing the spike-triggered average [22], and separating the spike-triggered average into its spatial and temporal component by singular-value decomposition. The spatial component was then fitted by a two-dimensional Gaussian function and its diameter determined as the diameter of an equivalent circle with the same area as contained within the 1-σ contour of the Gaussian. Similarly, temporal filters were obtained by computing the spike-triggered average under full-field Gaussian white-noise stimulation (updated at 50 Hz, 30% contrast). Peak amplitudes and latencies were determined from the spike-triggered average.

To test for direction selectivity, the retina was stimulated with moving square-wave gratings (100% contrast, 350 μm bar width, 1400 μm/s speed) in 8 different directions. From the average firing rates *f*_θ_ for the different motion directions θ, we calculated a direction selectivity index (DSI) as the magnitude of the normalized vector sum: *DSI =* |∑_θ_*f*_θ_e^iθ^|/∑_θ_*f*_θ_ [23, 24]. The DSI yields values between zero and unity, with larger values indicating stronger tuning for motion direction. In this study we defined ganglion cells with DSI>0.2 as direction-selective [25].

### Statistics

Statistical analysis between genotypes was carried out using the two-tailed unpaired t-test with Welch correction. For the multi-electrode array recordings, properties of ganglion cells from KO and WT mice were evaluated by a Wilcoxon rank-sum test. Differences in distributions of direction-selectivity were assessed by a Kolmogorov-Smirnov test. *, p≤0.05; **, p≤0.01; ***, p≤0.001.

## Results

### NL3 is localized at inhibitory postsynapses of the retina

**Fig 1:**
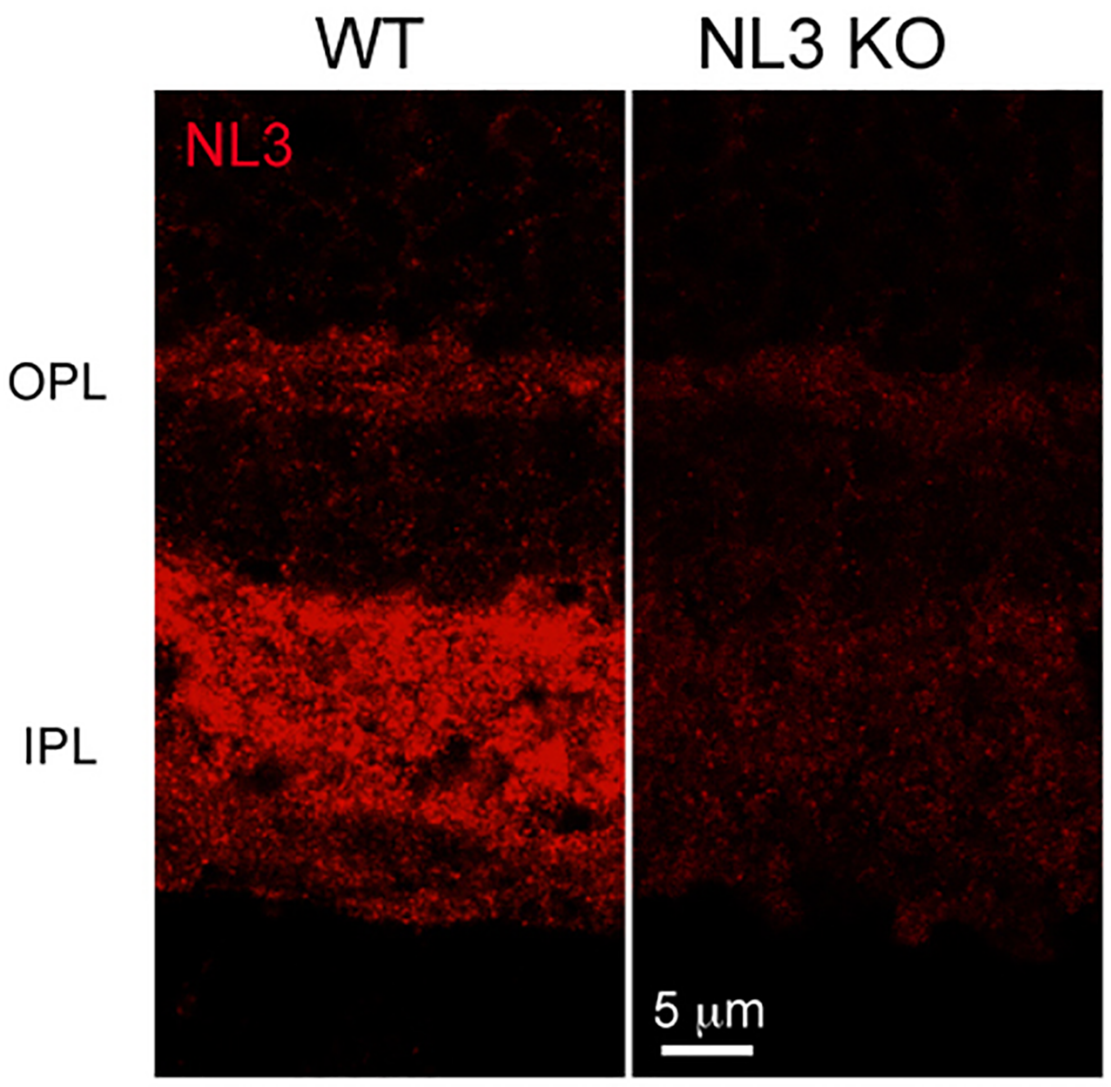
Expression of NL3 in the mouse retina. NL3, detected with an isoform-specific antibody, is robustly expressed at the inner synaptic layer of the WT retina. Accordingly, only faint background labeling is observed when the same antibody is applied on the retina of the corresponding NL3 KO. OPL, outer plexiform layer; IPL, inner plexiform layer.

**Fig 2:**
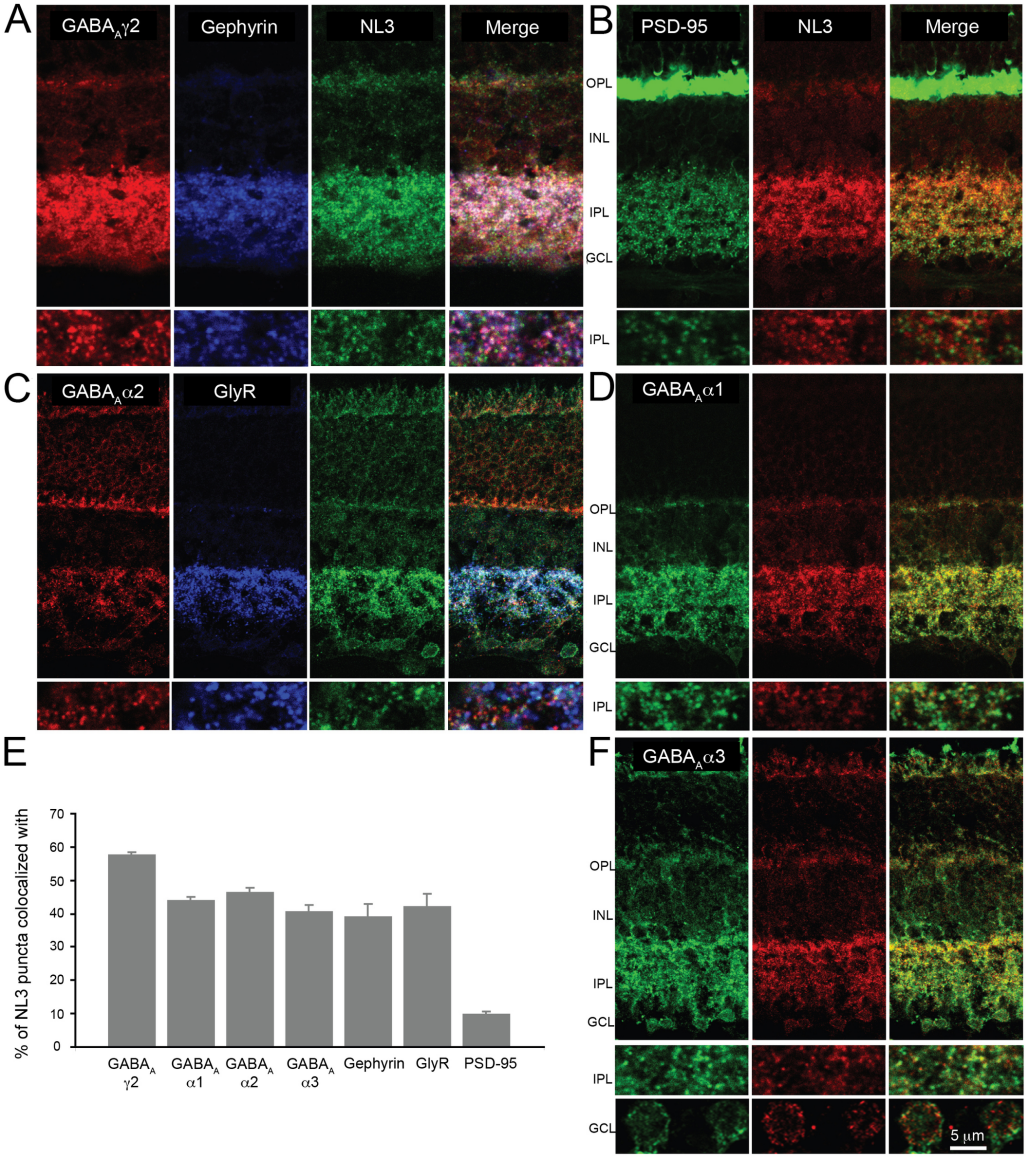
Localization of NL3 in the mouse retina. To ascertain the distribution of NL3 at retinal synapses, co-immunolabelings with excitatory (B) and inhibitory (A, C, D, F) postsynaptic markers were carried out. NL3 essentially did not associate with the excitatory postsynaptic protein PSD-95 (B, E); it colocalized extensively with the ubiquitous GABA_A_γ2 receptor marker (A, E) and equally well with GABA_A_α1, α2 and α3 receptor subsets, suggesting its association with diverse retinal GABA_A_ receptor subtypes (C-F). NL3 was also frequently observed together with glycine receptors (GlyR, labeled with a pan-GlyR antibody) (C, E). Plots in E represent true colocalization estimates after subtraction of random associations (see Methods). N = 3 animals and at least 4 image sections analyzed per staining. Plots represent mean ± SEM. OPL, outer plexiform layer; INL, inner nuclear layer; IPL, inner plexiform layer; GCL, ganglion cell layer.

A previously characterized NL3-specific antibody [1] was used to assess NL3 distribution in the mouse retina. It yielded a punctate labeling at the outer and inner plexiform layers (OPL and IPL) of the retina (Figs 1 and 2). A very faint residual background signal was visible in the KO retina sections (Fig 1, right panel) with the fixation and imaging conditions used, conditions which were necessary to allow the optimal detection of other proteins of interest (see Methods).

Co-immunolabelings for NL3 and different landmark postsynaptic proteins were carried out to characterize the synaptic localization of retinal NL3 puncta (Fig 2). NL3 essentially did not localize at presumptive excitatory postsynapses, identified by labeling for the excitatory postsynaptic protein PSD-95 (Fig 2B): only ~6% of NL3 puncta were found to overlap with PSD-95 clusters (Fig 2E).

Instead, NL3 associated prominently with inhibitory postsynaptic proteins. Shown in Fig 2A is the co-distribution of NL3 with the inhibitory postsynaptic scaffold protein gephyrin and the ubiquitous GABA_A_ receptor subunit GABA_A_γ2 [16, 26]. About 40% of NL3 puncta colocalized with gephyrin, whereas ~55% associated with GABA_A_γ2 (Fig 2E).

In view of this extensive overlap with GABA_A_ receptors, we further investigated the association of NL3 with specific GABA_A_ receptor subsets (containing either the GABA_A_α1, GABA_A_α2 or GABA_A_α3 subunits), which have been shown to occupy distinct non-overlapping retinal postsynapses [27, 28]. Upon co-labeling, it appeared that NL3 puncta associated equally with all three populations of GABA_A_ receptors in the retinal IPL (Fig 2C, 2D and 2F, quantified in Fig 2E). NL3 puncta were also observed around the soma of retinal ganglion cells (Fig 2C, 2D and 2F), and associated at times with GABA_A_ receptor clusters encircling the soma of ganglion cells (Fig 2F).

Interestingly, NL3 also co-distributed with glycine receptor (GlyR) clusters (Fig 2C), with ~40% of NL3 puncta colocalizing with receptor clusters detected with a pan-GlyR antibody (Fig 2E).

In summary, NL3 is essentially located at inhibitory postsynapses of the IPL, and colocalizes with both GABA_A_- and glycine receptor clusters (see also S1 Fig).

### Loss of NL3 does not alter overall retinal architecture but specifically leads to a dramatic decrease of GABA_A_α2- containing receptor clusters in the retinal IPL

**Fig 3:**
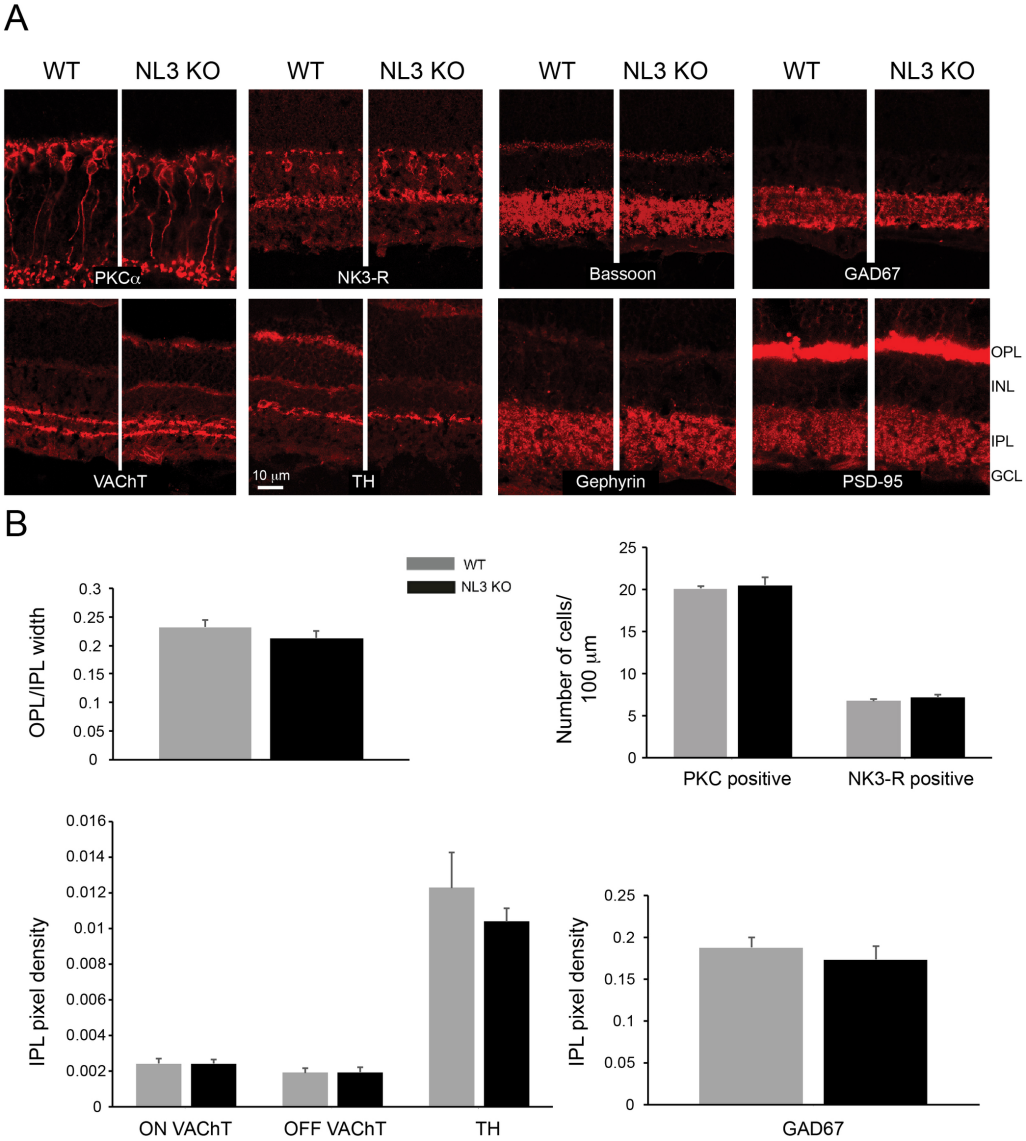
Architecture of the NL3 KO retina. Rod (PKCα) and OFF-cone (NK3-R) bipolar cells, cholinergic (VAChT), dopaminergic (TH) and GABAergic (GAD67) amacrine cells are similarly represented and organized in WT and NL3 KO retinae (A). The overall synaptic connectivity is intact in the absence of NL3, as illustrated by a comparable layout of ribbon synapses at the OPL and conventional synapses at the IPL (bassoon), and excitatory (PSD-95) and inhibitory (gephyrin) postsynapses in WT vs. NL3 KO retinae. Moreover, the relative thickness of the synaptic plexiform layers, as well as the number of PKC and NK3-R positive cells and the density of labeling for VAChT (both in the ON and OFF sublamina), TH and GAD67 were comparable across genotypes (N = 3 WT-KO littermate pairs, at least 5 images per sample) and point towards an intact retinal architecture of the NL3 KO (B). OPL, outer plexiform layer; INL, inner nuclear layer; IPL, inner plexiform layer; GCL, ganglion cell layer.

To assess the integrity of the retina in the absence of NL3, landmark cellular and synaptic proteins were labeled for and their distribution in WT and NL3 KO retinae compared (Fig 3). Rod bipolar and OFF-cone bipolar cell populations were visualized by PKCα [29] and Neurokinin 3 receptor (NK3-R) [30] labelings. The GABAergic amacrine cell population was labeled with GAD67 [29, 31] (Fig 3; GAD65 also yielded comparable staining patterns in WT and NL3 KO, not shown). The cholinergic and dopaminergic subsets of GABAergic amacrine cells were compared by labeling for vesicular acetylcholine transporter (VAChT) [32] and tyrosine hydroxylase (TH) [29]. Ribbon synapses at the outer plexiform layer and inhibitory synapses at the IPL were labeled with the presynaptic protein bassoon [33, 34], and excitatory and inhibitory postsynapses were labeled with PSD-95 [35] and gephyrin [36]. From these labelings (Fig 3A) and quantification (Fig 3B) of the ratio of the width of the synaptic plexiform layers, density of amacrine labels (VAChT, TH, GAD67) and number of bipolar cells (PKC and NK3-R positive cells) we observed no difference in the structural organization of the NL3 KO retina compared to WT littermates (Fig 3).

**Fig 4:**
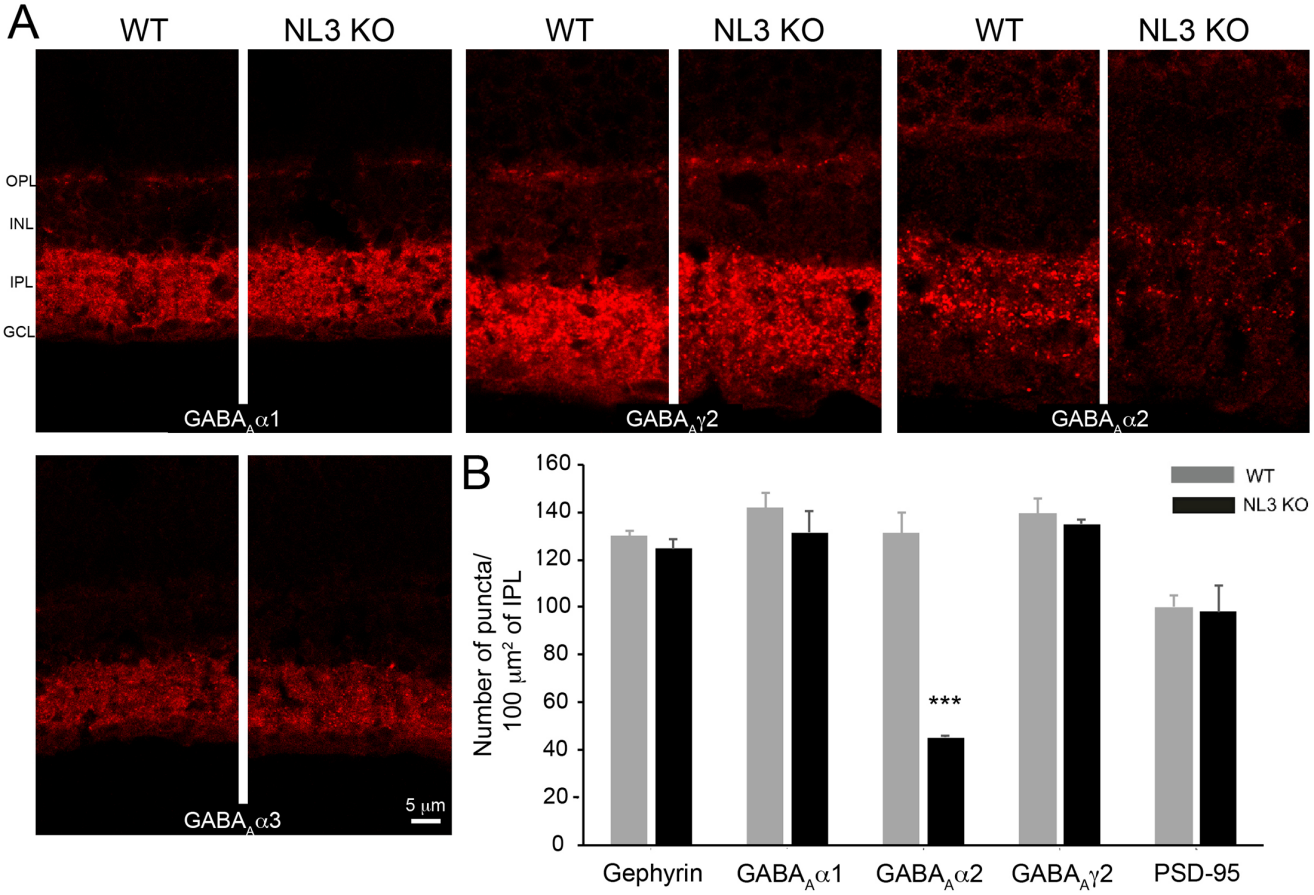
Distribution of GABA_A_ receptor clusters in WT and NL3 KO retina. To assess the integrity of the inhibitory postsynaptic compartment in the absence of NL3, WT and NL3 KO retinae were labeled for GABA_A_α1, α2, α3, and γ2 receptor subsets (A). A selective but dramatic reduction in the number of GABA_A_α2 receptor clusters was observed in the NL3 KO retina compared to WT (A, B). Of note, the number of the other GABA receptor subsets, Gephyrin and PSD-95 is comparable in WT and NL3 KO retinae. N = 4 WT-KO littermate pairs, at least 5 images per sample. OPL, outer plexiform layer; INL, inner nuclear layer; IPL, inner plexiform layer; GCL, ganglion cell layer.

**Fig 5:**
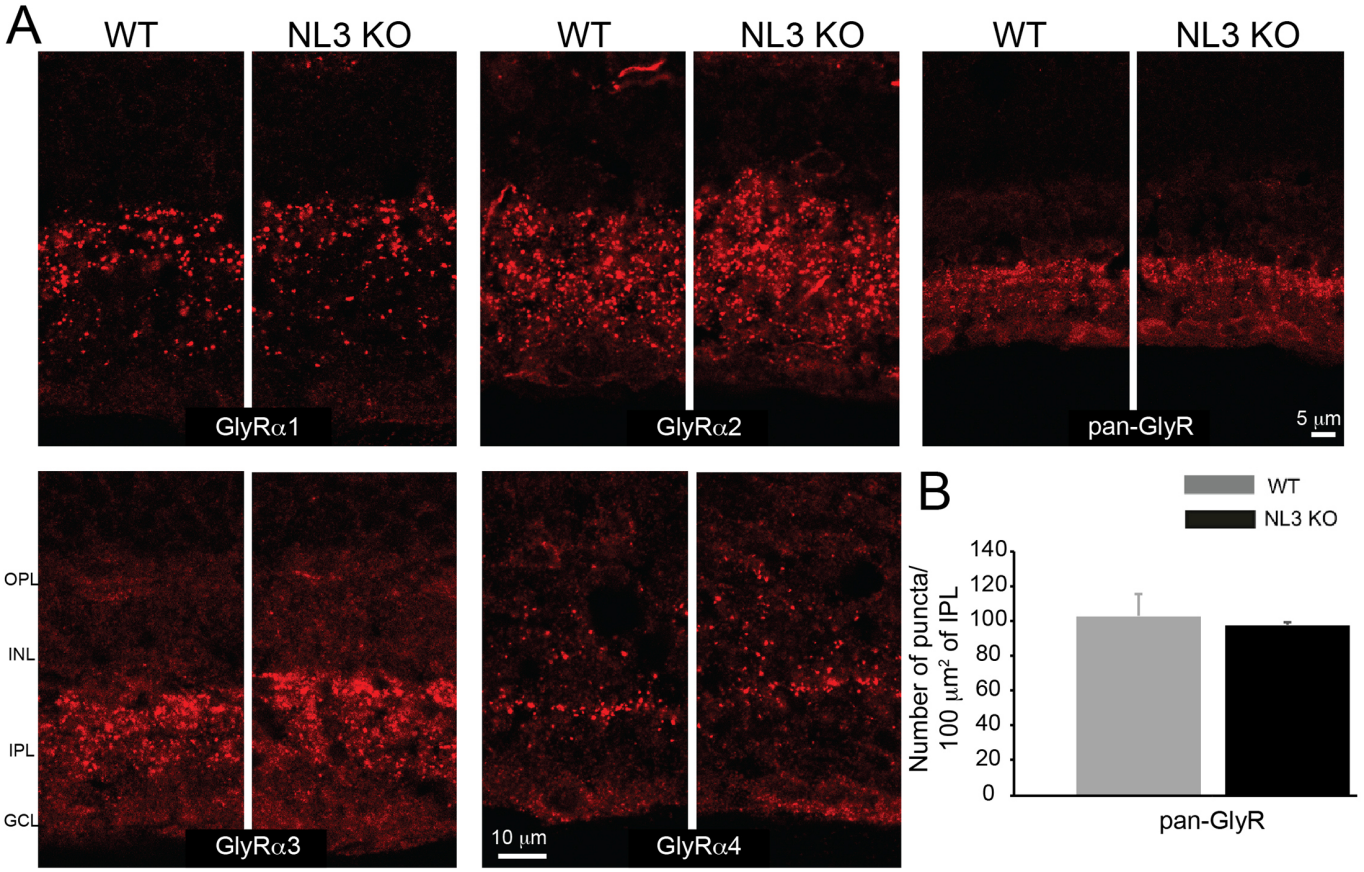
Distribution of glycine receptor clusters in WT and NL3-KO retina. Distinct glycine receptor subsets (containing GlyRα1 or α2 or α3 or α4) were immunolabeled in WT and NL3 KO retinae (A). Glycine receptor population was also labeled with a pan-glycine receptor (pan-GlyR) antibody. No alteration in the density or distribution of Glycine receptors was observed in the NL3 KO compared to control (A, B). N = 4 WT-KO littermate pairs, at least 5 images per sample. OPL, outer plexiform layer; INL, inner nuclear layer; IPL, inner plexiform layer; GCL, ganglion cell layer.

Since NL3 is essentially located at inhibitory postsynapses (Fig 2), we examined in particular the GABA_A_ and glycine receptor populations in WT and NL3 KO retinae. Diverse subsets of GABA_A_ receptors (bearing the ubiquitous γ2 or the GABA_A_α1, α2, α3 subunits; Fig 4) and glycine receptors (pan-GlyR label or bearing specifically the GlyRα1, α2, α3 or α4 subunits; Fig 5) were labeled and receptor cluster density quantified at the IPL of WT and NL3 KO (Figs 4B and 5B). With this analysis, we found a specific reduction in the number of GABA_A_α2 subunit-containing receptor clusters in the NL3 KO (WT_mean_ = 131.07 ± 8.56 puncta/100 μm^2^ of IPL; KO_mean_ = 45.21 ± 1.05 puncta/100 μm^2^ of IPL; n = 4 WT-KO littermate pairs; p = 0.01) (Fig 4A and 4B), whereas the other GABA_A_ receptor cluster densities remained comparable in both groups (Fig 4A and 4B). The numbers for Glycine receptors, gephyrin and PSD-95 were not altered in the NL3 KO retina (Figs 4B and 5B).

Taken together, these results showed that the loss of NL3 in the retina did not lead to an extensive alteration of the overall organization of the retina with respect to the main (excitatory) signal transmission pathway. The loss of NL3 also did not alter the modulatory glycinergic circuit. Instead, loss of NL3 led to a dramatic (~65%) reduction in the number of GABA_A_ receptor clusters containing the α2-subunit at both the ON and OFF laminae of the IPL.

We next went on to assess the functional consequences of NL3 deletion in the retina by measuring the responses of retinal ganglion cells (RGC)—the output cells of the retina—to light stimulation in WT and NL3 KO mice.

### Loss of NL3 impacts global retinal activity and RGC responses but does not massively impair direction selectivity

**Fig 6:**
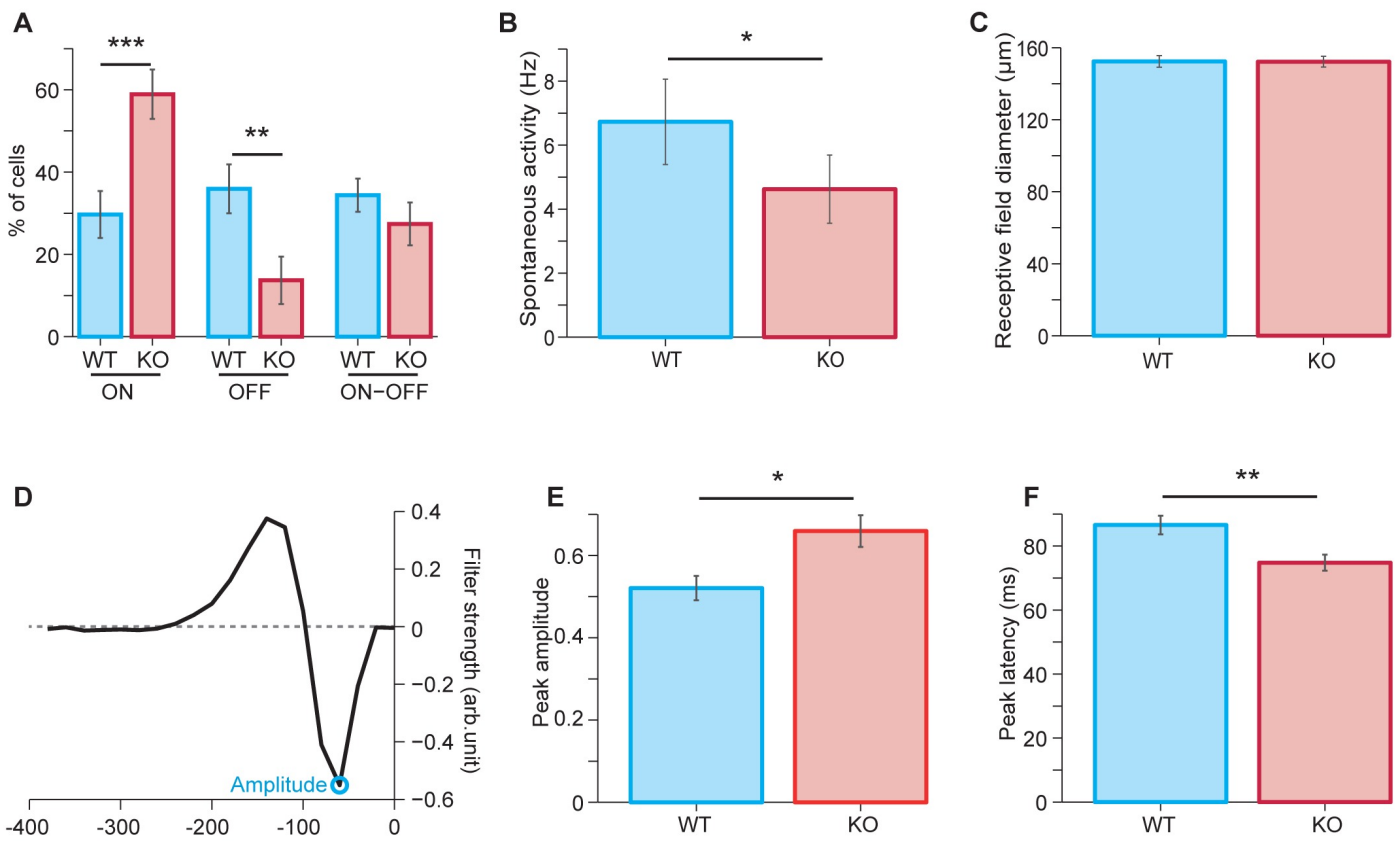
MEA recordings of RGC activity in WT and NL3-KO retina. The responses of RGCs to light stimulation were recorded on MEAs. The proportions of ON and OFF cells were respectively higher and lower in NL3 KO than in WT littermate retinae (**A**). Overall, NL3 KO RGCs had a lower spontaneous activity rate compared to WT RGCs (**B**); whereas their receptive field diameter was unchanged (**C**). Upon white-noise stimulation (**D-F**), filters from white-noise stimulation (example in **D** for a WT cell) had significantly larger peak amplitudes (**E**) and shorter peak latency (**F**) in NL3 KO retina compared to WT. Data from N = 3 WT- KO littermate pairs; WT = 64 cells (19 ON, 23 OFF and 22 ON-OFF); KO = 73 cells (43 ON, 10 OFF and 20 ON-OFF). Plots represent mean ± SEM. Data in (**B-F**) are pooled over all three response types.

RGC responses to light stimulation were assayed in NL3 KO and WT retina by multi-electrode array (MEA) recordings (Fig 6). RGCs are classified as ON, OFF or ON-OFF cells depending on their responses—occurring at light onset, offset or both. The proportion of ON cells was significantly higher in the NL3 KO retina compared to WT (WT: 30 ± 6%; KO: 58 ± 6%; p = 0.0003; Wilcoxon rank-sum test), whereas the relative number of OFF cells was significantly lower in the NL3 KO (WT: 36 ± 6%; KO: 14 ± 4%; p = 0.0012) and the proportion of ON-OFF cells remained unchanged (WT: 34 ± 6%; KO: 27 ± 5%; p = 0.19) (Fig 6A). Altogether, RGCs from the KO mouse had a significantly lower spontaneous activity as compared to WT cells (WT: 6.7 ± 1.3 Hz; KO: 4.6 ± 1.1 Hz; p = 0.028; Fig 6B). This decrease in spontaneous activity was most pronounced for ON cells (WT: 5.6 ± 2.5 Hz; KO: 3.5 ± 1.1 Hz) and ON-OFF cells (WT: 10.5 ± 1.1 Hz; KO: 7.1 ± 2.7 Hz), but appeared to be absent in OFF cells (WT: 4.0 ± 1.0 Hz; KO: 4.3 ± 2.8 Hz). By contrast, the size of the cells’ receptive fields did not differ significantly, neither for the entire population of recorded cells (WT: 152.39 ± 3.20 μm; KO: 152.23 ± 3.02 μm; p = 0.60; Fig 6C) nor for the three classes of ON, OFF, and ON-OFF cells separately (data not shown).

As a simple test for assessing whether visual signal processing is altered in NL3 KO retinas, we measured spikes under flickering light intensity by applying a white-noise stimulus. This allows us to compute a temporal filter for each recorded ganglion cell [22], which captures how the cell integrates visual information over time (WT example in Fig 6D). For the NL3 KO, the filters had larger peak amplitudes (WT: 0.52 ± 0.029 arb. units; KO: 0.66 ± 0.038 arb. units; p = 0.017; Fig 6E) and shorter peak latency (WT: 87 ± 3 ms; KO: 75 ± 2 ms; p = 0.0013 Fig 6F) as compared to the WT. This indicates that the NL3 KO does indeed affect the processing of visual stimuli, though more thorough investigations would be required to assess the exact nature of these alterations.

In view of the dramatic reduction in the number of GABA_A_α2-bearing synapses in the NL3 KO, one may hypothesize that retinal computations that are thought to rely on GABAergic signaling might be eliminated. The most prominent example of such function is given by direction-selective (DS) responses of specific types of ganglion cells [14, 37]. DS-RGCs respond strongly to stimulus motion in a specific direction, but are suppressed by GABAergic input from starburst amacrine cells (SACs) when the motion occurs in the opposite direction. Indeed, GABA_A_α2 clusters have been localized onto DS-RGCs in both the ON and OFF plexuses [38], as well as on the SACs that synapse onto these cells. We therefore tested whether direction selectivity could still be observed in NL3 KO RGCs or whether it was abolished.

**Fig 7:**
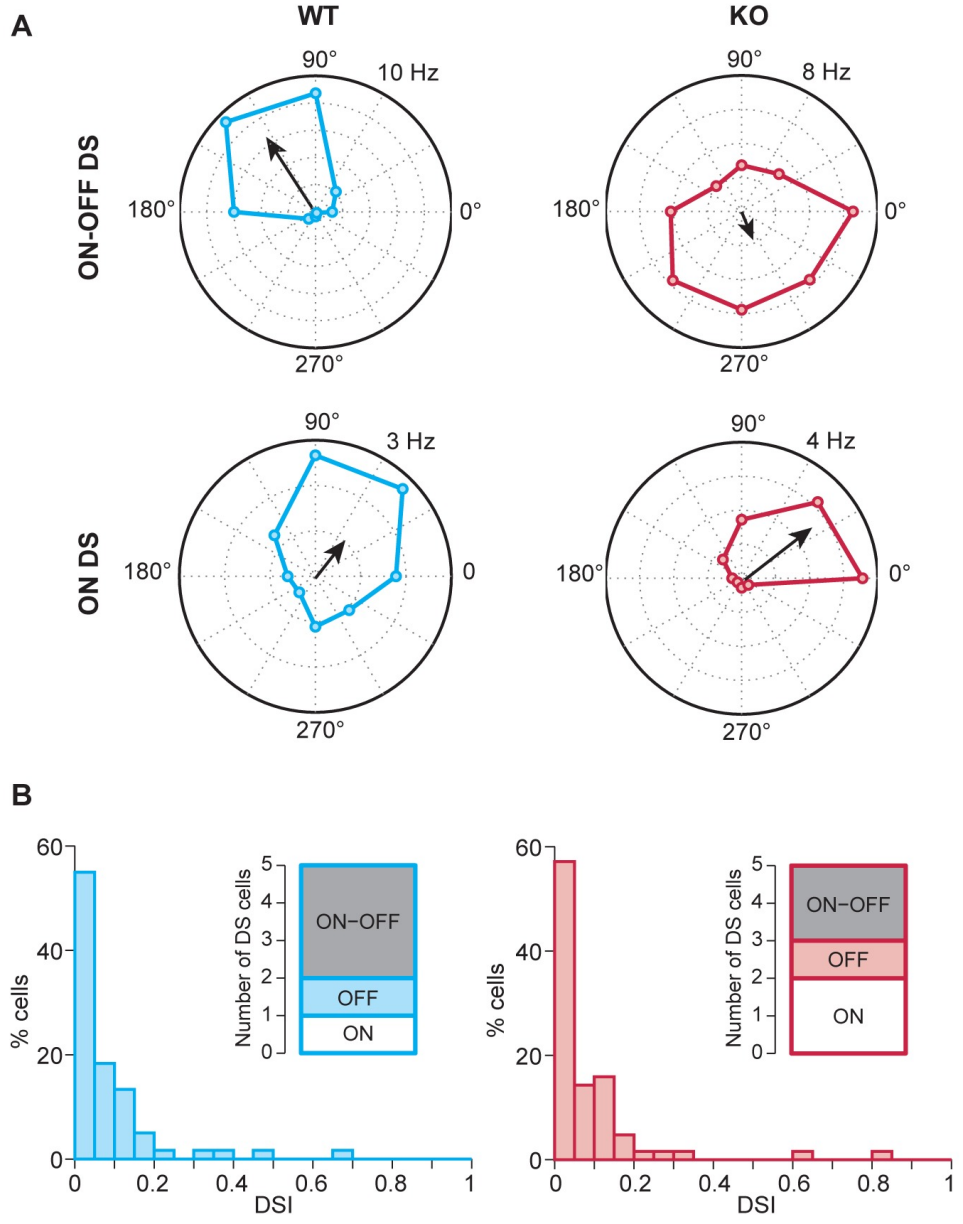
Features of DS-RGCs in WT and NL3-KO retina. Polar plots illustrate tuned responses of ON-OFF and ON DS-RGCs in WT and NL3 KO retinae (**A**). Direction-selectivity indices (DSI) of the cells recorded in WT and NL3 KO retinae showed a similar distribution pattern across genotypes, and the same numbers of DS-cells were observed in both genotypes (**B**).

We recorded ganglion cell responses under moving gratings with different directions of motion and assessed the cells’ direction preference by computing a direction selectivity index (DSI; see Methods). In both WT and NL3 KO, we identified 5 DS cells (Fig 7, as defined by DSI>0.2, see Methods). Fig 7A shows examples of the tuning of ON-OFF (top panel) and ON (bottom panel) DS-RGCs in NL3 KO and WT retinas. Both sets of DS cells contained at least one example from ON, OFF and ON-OFF DS cells (Fig 7B). There was no statistical difference between NL3 KO and WT retinas in the average DSI values (p = 0.70), in the distribution of the direction selectivity index (DSI) values (Kolmogorov-Smirnov test; p = 0.98) or in the numbers of identified DS RGCs (Fig 7B). Thus, direction selectivity was not abolished in NL3 KO retinas.

## Discussion

Neuroligins are among the first cell adhesion molecules identified to have a role in synapse formation and function [1, 2]. The distribution and roles of the four NLs have been extensively investigated in diverse neural networks using immunohistochemistry, functional analyses and comparison of mice lacking one or more NL isoforms to WT mice [1, 6–8]. These studies have revealed that NLs operate at specific postsynaptic connections: NL1 has a preferential localization at excitatory synapses [39] and controls the function of glutamatergic synapses via NMDA receptors in the hippocampus [40–42] and the striatum [43]; NL2 is widely expressed at inhibitory GABAergic synapses [15, 44–46], at cholinergic [47] and dopaminergic [48] synapses and modulates GABAergic synapse receptor aggregation and function in the retina [15], hippocampus [40, 49–51] and amygdala [52]; NL4 is localized at inhibitory synapses of retina, brainstem and spinal cord [9] and regulates glycinergic synapse receptor clustering and function in the retina [9] and GABAergic synapse function in the hippocampus [10].

Less is known about the synaptic specificity and role of NL3. In the present study, we took advantage of the well-identified stratified synaptic subsets of the mouse retinal plexus to determine NL3 distribution, and of the NL3 deletion-mutant (KO) mouse to assess its function *in vivo* using MEA recordings of RGCs. We found that more than 90% of NL3 puncta in the retinal IPL localize at inhibitory postsynapses, with a slight preference for GABA_A_-ergic over glycinergic postsynapses (Fig 2). Interestingly, NL3 has previously been reported at both glutamatergic and GABAergic postsynapses of cultured hippocampal neurons [11], and in the cerebellum where it was detected at different subpopulations of glutamatergic and/or GABAergic synapses depending on the layer scrutinized [8]. Altogether, these observations underscore the importance of extensive reporting of NL distribution and function to understand their specific contribution to synaptic architecture and neurotransmission, particularly considering that the four NL isoforms are highly homologous, very ubiquitous, and can form heterodimers [53].

In line with NL3 being strongly associated with populations of inhibitory synapses in the retina, we studied synaptic defects associated with NL3 deletion, and show that the absence of NL3 induces a dramatic two-thirds reduction in the density of GABA_A_α2 receptor puncta in the retinal IPL (Fig 4). The distributions of other GABA_A_ and Glycine receptors subunits were not massively altered in the NL3 KO (Figs 4 and 5). As a comparison, the absence of NL2 yielded specific decrease in the numbers of GABA_A_α3 and GABA_A_γ2 containing receptors, but not of GABA_A_α2-bearing receptors, in the retinal IPL [15]. Taken together, these data underline a clear specificity of each NL isoform in governing the clustering of different populations of postsynaptic receptors/components. Beyond these alterations, our further detailed investigation of the retinal circuit did not reveal any global reorganization of cell or synapse types in the absence of NL3 (Fig 3). Bipolar (rod and OFF cone) cell populations, amacrine cell subpopulations and pan-excitatory and inhibitory synapse densities were preserved in the NL3 KO retina (Figs 3 and 4). As a comparison, no massive network alterations have been observed within the retina of the NL2 KO either [15], aside from a putative compensatory alteration of the glycinergic circuit, or in the retinal organization of the NL4 KO [9]. Similarly, initial studies in the cortex, hippocampus and brainstem of the NL3 KO failed to uncover significant changes in excitatory or inhibitory transmission or synapse density [6]. More generally, it appears that mice deficient for single NL exhibit relatively subtle deficits and require focused investigation to be unraveled. Two studies of the NL3 KO mouse published during the preparation of this manuscript have revealed: 1) ectopic formation of excitatory synapses in the cerebellum, [8] as well as subtle alterations in excitatory transmission and mGluR1-mediated signaling [8] and 2) subtle alteration in inhibitory transmission and tonic endocannabinoid signaling in the hippocampus [12]. In zebrafish retina, mGluR1 is expressed at ON mixed rod/cone bipolar cell dendrites innervating rod and cone photoreceptor terminals [54]. There, the presence of mGluR1α is consistent with a role in retrograde endocannabinoid suppression [54]. Although NL3 in the retina is predominantly associated with inhibitory circuits at the IPL, its expression in the OPL (Fig 1) may be consistent with an additional role of NL3 in endocannabinoid signaling in the mouse retina via mGluR.

Finally, we investigated the functional consequences of NL3 absence on global retinal activity by recording RGCs activity via MEAs upon application of light flashes and white noise stimulation (Fig 6). The receptive field diameters of RGCs were comparable in WT and NL3 KO MEA recordings (Fig 6), suggesting that the absence of NL3 did not alter RGC dendritic coverage. Yet in the absence of NL3, ON RGCs were significantly more often encountered, and OFF RGCs less often compared to WT, whereas ON/OFF cells were observed in similar numbers (Fig 6). This suggests that either: 1) lamination (and hence connectivity) of the RGCs could be altered in the NL3 KO, resulting in more ON and less OFF RGCs; this could be tested in the future by labeling distinct RGC subpopulations (e.g. with SMI-32 [55]); or 2) alterations of direct or indirect inputs to RGCs in the NL3 KO may result in populations of ON and OFF RGCs that respond differently to light in the absence of NL3. Since the major bipolar cell circuits upstream of the RGCs seem to be preserved in the NL3 KO (Fig 3), we can speculate that changes in synapse architecture alone (Fig 3) could suffice to trigger such functional changes.

It has been reported that the total loss of GABA_A_α2 containing receptors massively impairs the response of direction-selective RGCs in the corresponding GABA_A_α2 KO mouse [38]. Interestingly, we report here instances of ON DS, OFF DS and ON-OFF DS cells (Fig 7, n = 5 cells in each group), in spite of a large (two-third) decrease in IPL GABA_A_α2 clusters numbers (Fig 4) caused by NL3 deletion. Thus direction selectivity is not entirely abolished in NL3 KO retinas. One explanation might be that the ~35% of GABA_A_α2 receptor clusters remaining in the IPL (Fig 4) in the absence of NL3 may be sufficient to preserve DS responses as seen in Fig 7. Alternatively, DS responses in the retina may not be entirely dependent on GABAergic inhibitory mechanisms, as suggested by a previous study showing that direction-selectivity can be implemented by excitatory mechanisms when GABA release from presynaptic starburst amacrine cells is disrupted in the mouse retina [56]. While the purpose of our analysis of direction selectivity was to test whether direction selectivity was still present or abolished in NL3 KO retinas, the small number of encountered DS cells in both WT and KO retinas prevented us from investigating subtle changes of RGC DS responses or of the DS circuitry. Future studies, perhaps using larger multi-electrode arrays [57] or targeted recordings of DS ganglion cells [58] will be needed to dissect out the different mechanisms contributing to retinal DS responses including NL3-driven synaptic organizations and unravel if indeed NL3 contributes towards shaping a yet unknown aspect of DS RGC responses.

Spontaneous activity and white-noise responses of RGCs did reveal alterations in the NL3 KO compared to control. We hypothesize that NL3-driven alterations in synaptic connectivity, in particular linked to specific subsets of GABA_A_ receptors, may impair direct inhibitory inputs onto RGC dendrites in the retinal IPL, yielding an increased peak amplitude of the RGC white noise filters (Fig 6); for example, the subset of NL3 puncta associated with GABA_A_ receptors around the soma of RGCs might constitute a specific perisomatic input to RGCs, whose function will have to be clarified in future studies. NL3-driven alterations may also impair a serial inhibitory network at the retinal IPL, resulting in an increased inhibitory drive onto RGCs which could account for the reduction in spontaneous activity of the RGCs in the NL3 KO and reduced peak latency of NL3 KO RGC filters to white-noise stimulation (Fig 6), similar to observations of reduced RGC activity upon a loss of GlyRα3 receptor synapses [59]. Of note, abolishing NL2, important for maintenance of GABA_A_α3/γ2 synapses in the inner retina, leads to increased baseline firing rate of NL2 KO RGCs compared to control and reduced amplitude and latency of NL2 KO RGCs to white-noise stimulation compared to control [15]. Deleting NL4 from the retina significantly reduces the density of IPL GlyRα1 receptor clusters, but does not affect baseline firing rate or peak amplitude of RGC responses as assayed by MEA recordings [9]. Biphasic STAs from NL4 KO RGCs however showed a shorter latency of the inflection point, indicating subtle yet distinctive effects on the coding capabilities of NL4 KO RGCs [9]. Taken together, observations from NL deficient retina underscore an isoform-specific role for the maintenance of inhibitory receptor populations and retinal circuit activity. Moreover measures of visual acuity and contrast sensitivity show selective perturbations among NL deficient animals, underscoring an isoform-specific contribution towards visually guided behaviors [9]. Future studies dissecting each of the inhibitory circuits (direct and serial) would be important to delineate the contribution of NLs to these postsynaptic populations and more importantly, to uncover the role of each of these motifs of IPL inhibition in shaping RGC responses and guiding visual behavior.

## Supporting information

S1 Fig NL3 localization with GABA_A_α2 and GlyR synapses. Co-labeling of NL3 with GABA_A_α2 (**A**) and NL3 with glycine receptors (GlyR) labeled with a pan-GlyR antibody (**B**). Triple labeling of these markers is shown in Fig 2C. NL3 puncta are more tightly associated with GABA_A_α2 than GlyR clusters. OPL, outer plexiform layer; INL, inner nuclear layer; IPL, inner plexiform layer; GCL, ganglion cell layer.
(TIF)
