# Supplementary figures and images for: Loss of Neuroligin3 specifically downregulates retinal GABAAα2 receptors without abolishing direction selectivity
Source: PLoS One. 2017 Jul 14;12(7):e0181011. doi: 10.1371/journal.pone.0181011 (PMC5510863; doi:10.1371/journal.pone.0181011)

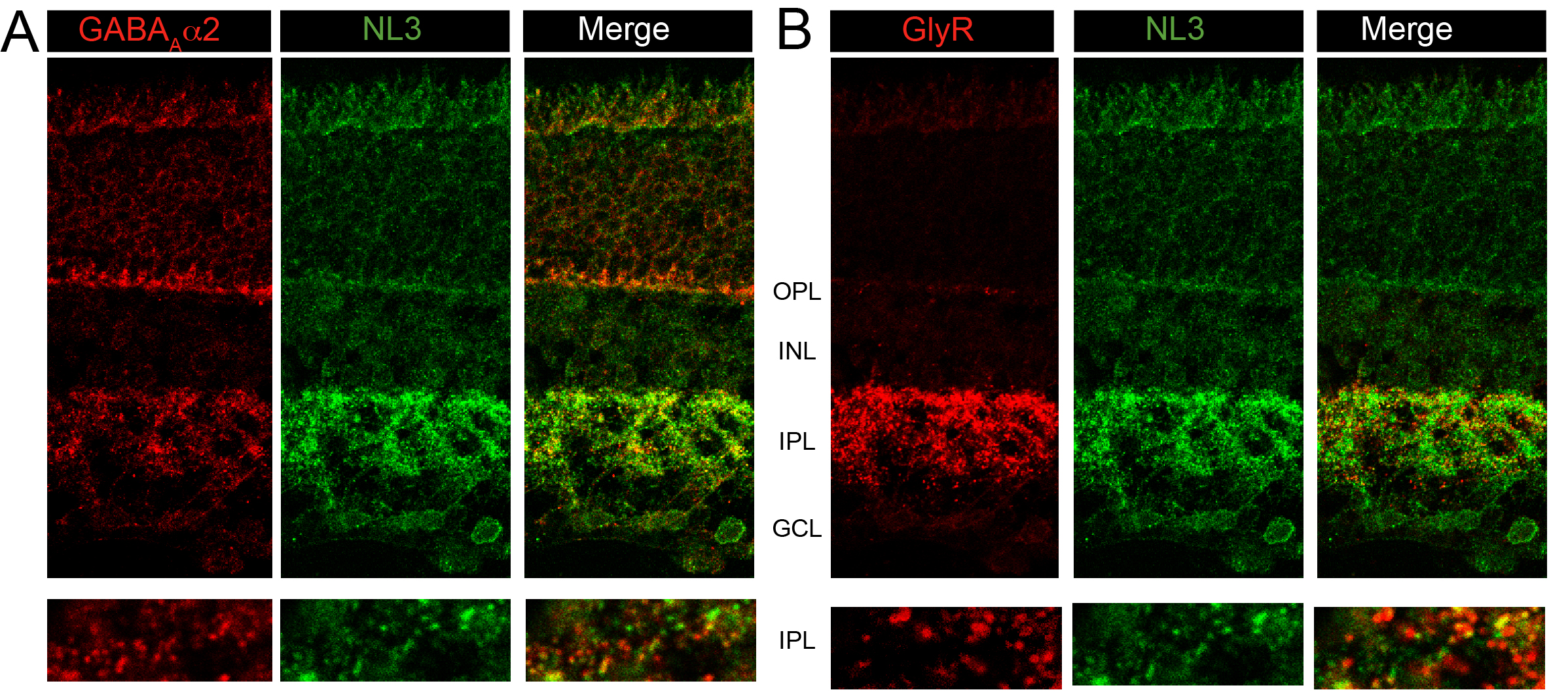

Supplement: S1 Fig — Co-labeling of NL3 with GABAAα2 (A) and NL3 with glycine receptors (GlyR) labeled with a pan-GlyR antibody (B). Triple labeling of these markers is shown in Fig 2C. NL3 puncta are more tightly associated with GABAAα2 than GlyR clusters. OPL, outer plexiform layer; INL, inner nuclear layer; IPL, inner plexiform layer; GCL, ganglion cell layer. (TIF) [file pone.0181011.s001.tif]
